# Case Report: Long-term survival of refractory high-grade B-cell lymphoma with MYC, BCL2, and BCL6 rearrangements through glofitamab monotherapy consolidated by ASCT

**DOI:** 10.3389/fimmu.2026.1857464

**Published:** 2026-06-30

**Authors:** Fang Fang, Jing Ni, Hong Zhao, Wuhan Hui, Xuejing Sun, Dongmei Zou, Wanling Sun

**Affiliations:** 1 Department of Hematology, Xuanwu Hospital, Capital Medical University, Beijing, China; 2 National Clinical Research Center for Geriatric Diseases, Beijing, China

**Keywords:** autologous stem cell transplantation (ASCT), bispecific antibody, early progression, high-grade B-cell lymphoma, immune

## Abstract

Patients with high-grade B-cell lymphoma (HGBCL), who are in early relapse or primary refractory, always have poor outcomes. Even intensive chemotherapy or CAR-T cell therapy is not always the best solution. Here is a case of refractory high-grade B-cell lymphoma with MYC, BCL2, and BCL6 rearrangements (triple-hit HGBCL), who achieved complete metabolic remission (CMR) following six cycles of glofitamab monotherapy induction and followed by autologous stem cell transplantation (ASCT) consolidation. The patient has achieved a sustained CMR and a progression-free survival (PFS) of 25 months (ongoing) from progression. Hepatitis B virus (HBV) seroconversion occurred during ASCT before stem cell engraftment, and was effectively suppressed with entecavir. Immune function was monitored in our case by flow cytometry. Downregulation of PD-1 expression on T cells and a reduced proportion of regulatory T cells (Tregs) were observed, consistent with fully activated immune function, which may explain why the patient responded rapidly and maintained durable efficacy. Immune analysis also exhibited B cell subset exhaustion and a disrupted naïve/memory T cell ratio which suggested an immunosenescence phenotype. It is speculated that an over activation of immune function promotes immunosenescence following bispecific antibody therapy, this needs attention. This case highlights the potential of glofitamab induction followed by ASCT consolidation to achieve durable remission in aggressive triple-hit HGBCL. The immune function after bispecific antibody treatment should be monitored and needs further investigation.

## Introduction

Diffuse large B-cell lymphoma/High-grade B-cell lymphoma with MYC and BCL2 rearrangements (DLBCL/HGBCL-MYC/BCL2) is an aggressive B-cell lymphoma characterized by chromosomal aberrations with breakpoints at both MYC and BCL2 loci (1). Triple-hit high-grade B-cell lymphoma (HGBCL) is referred to as DLBCL/HGBCL-MYC/BCL2 with an additional BCL6 rearrangement (2). It is associated with a high refractory or relapse rate after standard CHOP-like chemo-immunotherapy. Several studies suggest that intensive first-line chemotherapy can improve progression-free survival (PFS) and overall survival (OS) in patients with advanced-stage (Ann Arbor III/IV) DLBCL/HGBCL-MYC/BCL2 (3). However, not all patients in good general condition can tolerate intensive chemotherapy (4). And still some patients are primary refractory (5). Once patients are in early relapse or primary chemo-resistant, the prognosis is dismal.

The Chimeric Antigen Receptor T-cell (CAR-T) therapy targeting CD19 has shown promising efficacy in chemotherapy-resistant Large B-cell lymphoma (6–8). However, the median time from enrollment to the CAR-T cell infusion is 51 days (9). Patients with relapsed/refractory (R/R) DLBCL/HGBCL-MYC/BCL2 always progress rapidly; as a result, up to 23.8% of patients die before CAR-T cell infusion. Among patients who received CAR-T infusion, only 1/3 of patients obtained a long-term response (10). In addition, subgroup analysis revealed that OS was only 6.6 months in patients with DLBCL/HGBCL- MYC/BCL2, which was significantly lower than that of patients with HGBCL-NOS (18.5 months), HGBL-NOS with MYC and BCL6 rearrangement (13.6 months), or non-HGBCL (11.8 months) (11). Salvage autologous hematopoietic stem cell transplantation (ASCT) also cannot overcome the poor prognosis (12).

Glofitamab, is a readily available bispecific antibody without a waiting period and has a manageable safety profile, making it suitable for highly aggressive lymphoma. In clinical trials, glofitamab monotherapy demonstrated long-term efficacy, with a median CR duration of 26.9 months across all patients (13), but its efficacy in real-world settings is not promising. For patients with R/R DLBCL/HGBCL-MYC/BCL2, the median PFS is 2.5 months, and the median OS is only 4.1 months (14).

Here is a case with triple-hit HGBCL in early progression. A complete metabolic remission (CMR) was achieved with glofitamab monotherapy induction, followed by ASCT consolidation. A long-term survival with a PFS of 25 months from progression has been achieved, and a CMR status has been sustained. In addition, immune function was monitored in this case by flow cytometry.

## Case presentation

**Figure 1:**
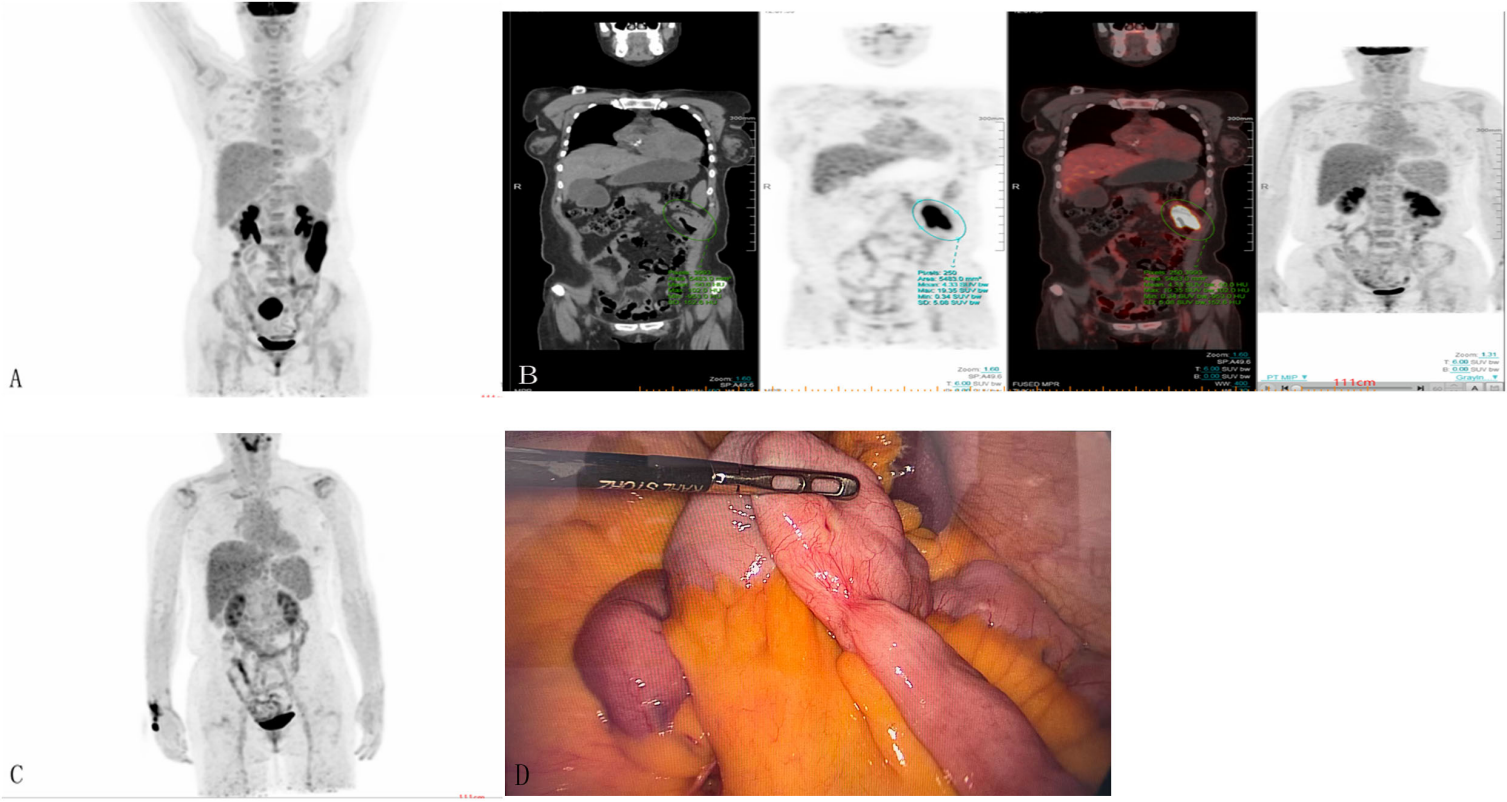
**(A)** The PET-CT scan at diagnosis (SUVmax 16.2). **(B)** The PET-CT scan at disease progression (SUVmax 20.33). **(C)** The last PET-CT scan for response evaluation. **(D)** A stenotic segment of the small intestine was confirmed by laparoscopic examination.

A 50-year-old woman presented with abdominal pain and abdominal distension two months before seeking medical attention, which was aggravated by eating and relieved by vomiting. Then she underwent gastroscopy, which revealed multiple gastric body polyps, chronic superficial gastritis, and thickened, elevated jejunal mucosa (cancer)?. To further confirm the diagnosis, small bowel endoscopy and Positron emission tomography/computed tomography (PET/CT) were performed. Histopathological examination confirmed diffuse large B-cell lymphoma (DLBCL) in the jejunum. The immunohistochemistry findings showed that the malignant cells were positive for CD20, CD79a, BCL6, CD10, PAX-5, BCL2, c-MYC, and negative for CD3, MUM-1, P53, and EBV. The Ki-67 showed a proliferative index of 80%. Pathology results reported DLBCL (Germinal center B-cell-like subtype, double expression lymphoma). Next-Generation Sequencing (NGS) revealed the molecular subtype was TP53 mutation type with class I mutation in TP53 and CREBBP mutation. The PET-CT showed intestinal wall thickening in the jejunum and colon, multiple enlarged lymph nodes, and a mass located in the right pelvic region near the para-spinal region, with an elevated standard uptake value (SUV, SUV_max_ 16.2, maximum size of mass 49 × 39 ×51mm) (**Figure 1A**). Bone marrow biopsy did not reveal any lymphoma cells. Laboratory data showed that lactate dehydrogenase was normal (186 U/L). The Eastern Cooperative Oncology Group (ECOG) score is 2. The age-adjusted International Prognostic Index (IPI) score was calculated at 2. The patients’ final diagnosis was DLBCL (Hans’ algorithm: GCB subtype, Stage IV, Group A, aaIPI: high-intermediate risk). Fluorescence *in situ* hybridization (FISH) to detect MYC, BCL2, and BCL6 rearrangements was performed, but the results did not return when treatment was initiated. The patient received Pola-R-CHP (polatuzumab vedotin, rituximab, cyclophosphamide, epirubicin, and prednisone). During the first two-cycle therapy, the abdominal pain and distension gradually improved. However, FISH results showed that all c-MYC-, BCL-2-, and BCL-6-related gene translocations were positive, indicating triple-hit HGBCL. Then rituximab and high-dose methotrexate were administered as CNS prophylaxis. Upon readmission for the next cycle of chemotherapy, the patient reported abdominal pain worsening after consuming meat, relieved by defecation. Complete blood count (CBC) revealed microcytic hypochromic anemia. The PET-CT showed marked thickening of the left mid-abdominal small bowel wall with significantly higher SUV (SUVmax 20.33, **Figure 1B**) than at diagnosis. At the time of progression, the patient’s condition worsened quickly and the patient was barely able to eat and drink. Given the historical efficacy of CAR-T in triple-hit HGBCL and the manufacturing time (11), the patient prefers the readily available CD20 × CD3 T-cell-engaging bispecific antibody glofitamab, as second-line therapy. During glofitamab administration, the patient developed only grade 1 cytokine release syndrome (CRS) during cycle 1 and 2. No other significant adverse events were observed. The patient was treated with glofitamab for 6 cycles. A complete metabolic response was observed on PET-CT evaluation after 5 cycles of glofitamab treatment (**Figure 1C**).

**Figure 2:**
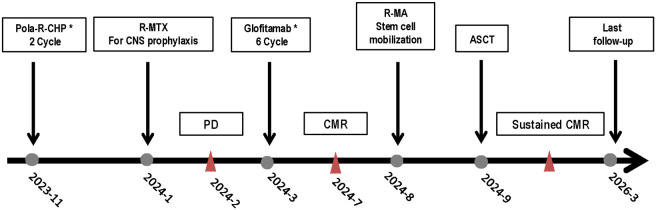
The therapy timeline in this case. Pola-R-CHP, polatuzumab vedotin, rituximab, cyclophosphamide, doxorubicin, and prednisone; R-MTX, rituximab, high-dose methotrexate; R-MA, rituximab, high-dose methotrexate, high-dose cytarabine; PD, progressive disease; CMR, complete metabolic response. ASCT, Autologous hematopoietic stem cell transplantation.

This patient is primary refractory to first-line therapy, which predicted a high failure rate. Previous reports displayed that those who attained CR after salvage therapy with sequential ASCT consolidation fare better outcomes (15). Since this patient is 50 years old without any comorbidities, has an ECOG score of 2, and has CMR status, ASCT proceeds as consolidation. She received rituximab combined with high-dose methotrexate and high-dose cytarabine as a stem cell mobilization regimen; peripheral blood mononuclear cells at 2.92×10^8/kg and CD34+ cells at 28×10^6/kg were collected. Carmustine, etoposide, cytarabine, and melphalan (BEAM) were selected as the conditioning regimen. On day +6, the patient developed a high fever. She received antimicrobial therapy with imipenem-cilastatin, vancomycin, and voriconazole, but the treatment proved ineffective. On day +11, the metagenomic NGS (mNGS) showed positivity for herpes simplex virus-1 (HSV-1) and hepatitis B virus (HBV). Although the mNGS revealed 110 sequence reads specific to HBV with high confidence, HBV DNA copies were negative by PCR. And HBV screen indicated seroconversion from anti-hepatitis B core antibody (anti-HBc) - negative to positive, with a marked increase in hepatitis B surface antibody (anti-HBs) titer, but negative hepatitis B surface antigen (HBsAg) and a normal ALT level. This patient underwent occult HBV infection and HBV seroconversion rather than HBV reactivation (16). The patient received intravenous immunoglobulin (IVIG) and acyclovir, along with oral entecavir. Following treatment, the patient’s temperature returned to normal. Platelet and neutrophil engraftment were achieved on day +10 and +12, respectively. One year after ASCT, a follow-up evaluation of PET/CT confirmed a sustained CMR status; however, the patient continued to complain of abdominal discomfort and occasional vomiting. An incomplete intestinal obstruction was suspected, resulting from intestinal local scarring at the primary lesion. Laparoscopic examination confirmed a stenotic segment of small intestine approximately 1 meter distal to the ligament of Treitz, and the narrowed segment was resected(**Figure 1D**). Postoperatively, the patient’s symptoms resolved. Pathological examination of the resected bowel showed chronic inflammation and no evidence of lymphoma infiltration. This patient achieved a long-term survival with a PFS of 25 months (ongoing). The therapy timeline is shown in **Figure 2**.

**Figure 3:**
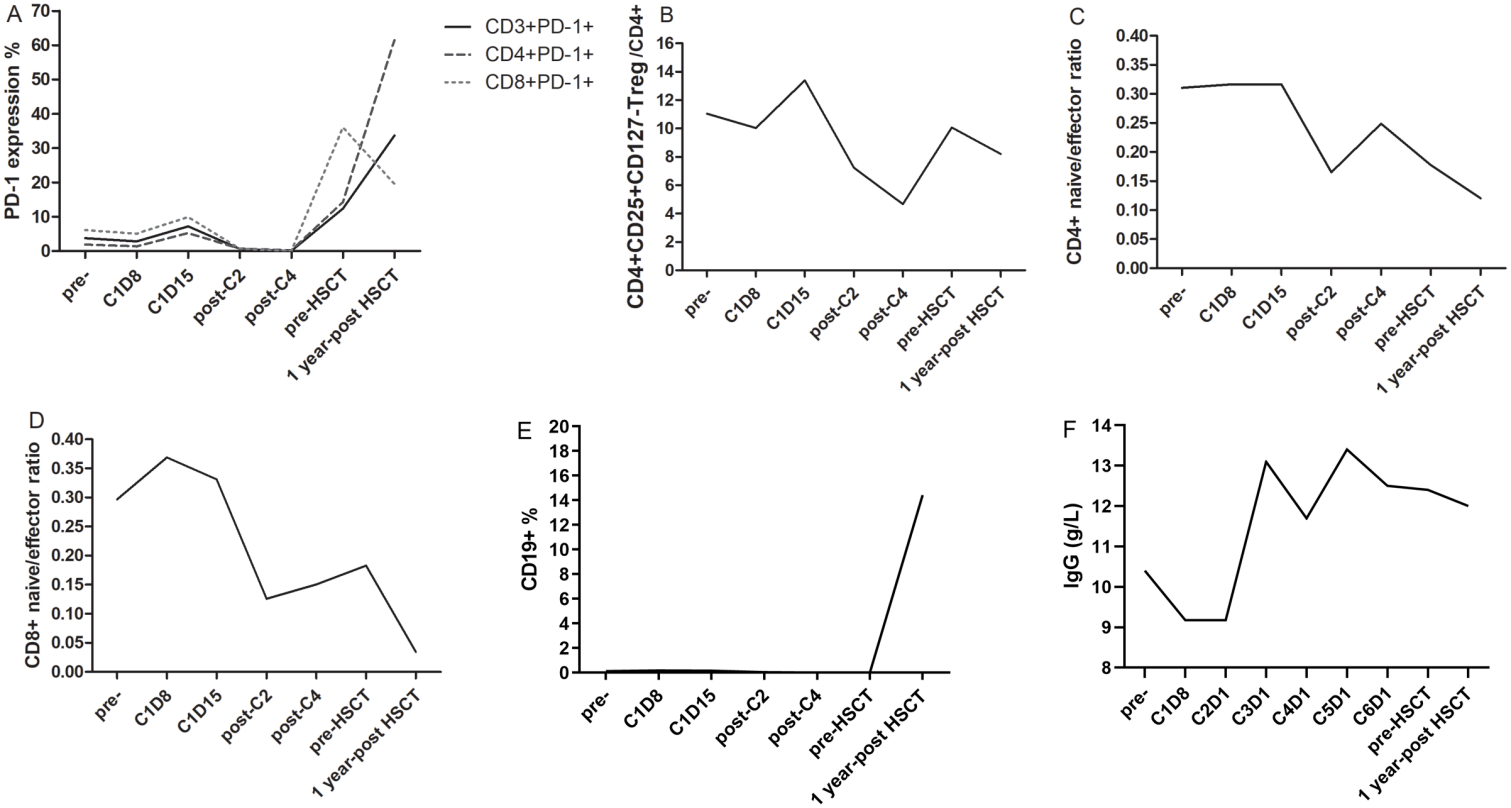
**(A)** PD-1 expression on CD3+T cells, CD4+T cells, and CD8+T cells during therapy. **(B)** The percentage of Regulatory T cells (Treg) in CD4+ T cells. **(C)** The ratio of CD4+ naïve T cell/effector T cell. **(D)** The ratio of CD8+ naïve T cell/effector T cell. **(E)** CD19+B cell percentage in peripheral blood. **(F)** IgG level during therapy.

The immune cell subsets in peripheral blood were detected by multi-parameter flow cytometry. It revealed that PD-1 expression on T cells (**Figure 3A**) and the proportion of CD4+CD25+CD127- regulatory T cells (Treg) (**Figure 3B**) were downregulated during six cycles of glofitamab. While naïve T cell subsets decreased, memory T cell subsets increased. The CD4+ and CD8+ naïve/memory T cell ratio decreased following glofitamab treatment (**Figures 3C, D**). Moreover, the B cell subset was exhausted during therapy (**Figure 3E**), but the IgG level remained within the normal range during treatment (**Figure 3F**).

## Discussion

DLBCL/HGBCL-MYC/BCL2 is a highly aggressive lymphoma. Once patients are in refractory or relapsed status, the median OS is 6.3 months (5). Long-term survival remains a challenge. Non-cross-resistant immune-chemotherapy and sequential ASCT consolidation produce a disappointing outcome (2). Targeted inhibitors combination result in excess treatment-related mortality and do not significantly improve PFS (17). Salvage allogeneic hematopoietic stem cell transplantation (allo-HSCT) is an option for R/R DLBCL/HGBCL-MYC/BCL2. However, it usually works in patients with chemo-sensitive disease, because only disease in remission after salvage chemotherapy allows space and time for graft-versus-lymphoma (2). For patients with chemorefractory disease, poor performance status, and an interval of less than 1 year after ASCT, allo-HSCT did not yield a satisfactory outcome (12). Allo-HSCT has only been suggested for those who attain CR after salvage high-dose chemotherapy. Currently, CAR-T cell therapy is recommended for patients with R/R Large B-cell lymphoma, but patients with R/R DLBCL/HGBCL-MYC/BCL2 progress quickly and may die before CAR-T cells are manufactured (18). And among patients with HGBCL who received CAR-T infusion, only 1/3 obtain a long-term response (10). DLBCL/HGBCL-MYC/BCL2 is a relatively chemo-resistant disease, but not immune-resistant. Glofitamab is a bispecific T-cell engager that targets CD20 on B cells and simultaneously binds CD3 on T cells, recruiting T cells to the cancer site, making it promising. Compared with CAR-T cells which have a long turnaround time and a risk of cell production failure, glofitamab is readily available without a waiting period and is more suitable for rapidly progressing lymphomas. In addition, the clinical toxicity of CAR-T cells would be a barrier for old and frail patients (19). Glofitamab has a manageable safety profile. Most patients reported grade I-II CRS during therapy. From an economic standpoint, CAR-T cell is high priced (20); glofitamab is a relatively cost-efficient choice. Therefore, for those with highly aggressive lymphoma like triple-hit HGBCL, or patients in frail condition who cannot tolerate CAR-T cell toxicity or cannot afford one dose of CAR-T cells, glofitamab would be a promising alternative.

In our case, the patient has achieved a PFS of 25 months (ongoing) since the progression. It displays definite efficacy of glofitamab in refractory triple-hit HGBCL and highlights the potential of glofitamab induction followed by ASCT consolidation to achieve durable remission. Downregulation of PD-1 expression on T cells and a reduced proportion of Tregs were consistent with fully activated immune function that may explain why the patient responded rapidly and maintained durable efficacy. Severe CRS is not common after glofitamab infusion. Vulnerability to infection is possibly a major concern. Lymphocyte subset analysis indicates B-lymphocyte exhaustion which may be responsible for infection proneness. The patient in our case suffered from HSV-1 infection and HBV seroconversion occurred during ASCT before stem cell engraftment which was effectively suppressed with medicine. In general, this regimen is safe and manageable. Thirdly, a possible immunosenescence phenotype was observed during the therapy. Whether an overactive immune function promotes immunosenescence following bispecific antibody therapy, small-sample cohort studies should be carried out in the future to verify this immune phenomenon. Due to limitations of a single-case report, the conclusions from our case should be applied cautiously and not be directly generalized to all patients with triple-hit HGBCL.

Glofitamab monotherapy in clinical trials showed promising long-term efficacy (13, 21, 22), but the real-world efficacy in relapsed/refractory HGBCL has been less encouraging. The median PFS is 2.5 months, and the median OS is only 4.1 months (14). Long-term PFS remains a challenge in R/R HGBCL. Previous reports have shown that even if refractory or early relapsed patients with myc-translocated DLBCL respond to salvage chemotherapy, they relapse after ASCT, and none of them obtain long-term survival (23). A recent case report showed relapse and refractory Burkitt’s lymphoma were treated with the glofitamab and polatuzumab vedotin (24). All three cases obtained a CMR after 2-5 treatment cycles. Then two of them underwent allo-HSCT as consolidation and remained CMR, while one of them received CD19-CAR-T cells as consolidation but progressed after 3 months. In our case, long-term survival and a sustained CMR status were achieved in patients with triple-hit HGBCL through glofitamab induction and ASCT consolidation. For those achieving remission with glofitamab, ASCT consolidation might be a wise choice to achieve a durable response. It seems reasonable that ASCT consolidation produces different outcomes after chemotherapy induction and bispecific antibody induction. ASCT is, in essence, similar to high-dose chemotherapy, whereas a bispecific antibody is an immunotherapy that actives T cells and drives them to the cancer site. The combination of ASCT and bispecific antibodies may exert a synergistic anti-lymphoma effect. The exact mechanism behind the synergistic effect, such as a deeper remission, or chemo-resistant lymphoma eradiation, remains under investigation.

In our case, glofitamab displays a manageable safety profile. CRS events were common but predominantly grade 1-2 (14). Besides CRS, infection is the most common side effect. Any-grade infection occurred in 40% of patients, and severe infections (grade≥3) in 20% (25). Viral infections were the most common cause of fatal infections. This patient experienced HSV-1 infection and HBV seroconversion with a normal ALT during HSCT, and the infection were effectively suppressed with antiviral therapy. During therapy, lymphocyte subpopulation analysis indicates B lymphocyte exhaustion, which may explain the vulnerability to infection. The B lymphocyte proportion in this patient returned to normal one year later after treatment completion. Because of the ASCT, the immune reconstitution following bispecific antibody therapy cannot be determined. Another study recently reported that B cell recovery was observed in 42% of patients by 12 months and in 86% by 18–24 months (26). During this period, hypogammaglobulinemia should be monitored, and IVIG replacement therapy is recommended when the IgG level is <4 g/L or when severe infections are present (27). IgG level in our case remains within a normal range, possibly attributed to the fact that long-lived plasma cells cannot be targeted by glofitamab.

Immune function was monitored by flow cytometry. Downregulation of PD-1 expression on T cells and a reduced proportion of Tregs were observed, consistent with fully activated T-cell function following glofitamab treatment. A murine myeloma model (22) revealed that the proportion of Treg cells decreased, and PD-1 expression on Treg cells rose, suggesting that the immunosuppressive function of Treg cells was weakened following BCMA-T-cell-engaging bispecific antibodies treatment. Other clinical studies have also confirmed that the enumeration and proportion of Treg cells are closely associated with therapeutic response (28, 29). Therapeutic removal of Tregs may convert non-responders to responders (28, 30). This patient exhibited a decrease in Treg numbers, which may explain why the patient responded rapidly to treatment and maintained durable efficacy. Naïve T cell and memory T cell imbalance is an important hallmark of immunosenescence (31, 32). Following glofitamab treatment, the lymphocyte subset also exhibited an immunosenescent phenotype, with a disrupted naïve/memory T-cell ratio. Therefore, it is speculated that an overactive immune system following bispecific antibody therapy may promote immune senescence. This phenomenon needs further attention and investigation.

## Data Availability

The original contributions presented in the study are included in the article. Further inquiries can be directed to the corresponding author.
